# Grit in Medical Education: Differing Perspectives of Residents and Mentors

**DOI:** 10.7759/cureus.8315

**Published:** 2020-05-27

**Authors:** Michael J Asken, Siddharth Goel, Isha Shrimanker, Michelle-Ashley Rizk, Nicholas Abourizk, Vinod Nookala

**Affiliations:** 1 Internal Medicine, University of Pittsburgh Medical Center Pinnacle, Harrisburg, USA; 2 Internal Medicine, Community Medical Center, Toms River, USA

**Keywords:** grit scale, resident, faculty, non-cognitive, burnout

## Abstract

Background

Increasing concerns about depression and burnout in residents have led to a recent focus on assessing “non-cognitive” traits in residents and residency applicants. One attribute that has received significant attention is grit, defined as trait-level perseverance and passion for long-term goals^. ^With an objective measure available, an important question is under what circumstances of administration is that measure reliable and accurate. The goal of this study was to ascertain whether internal medicine residents and their faculty mentors were congruent in their ratings of resident grit, or if not, how the ratings differed.

Methods

Subjects were internal medicine residents (N=42) at a community-based university-affiliated hospital internal medicine residency program. Near the end of the academic year 2019, residents completed the GRIT-S (short form). As each resident is assigned a mentor during their training, each resident’s mentor was also asked to complete the GRIT-S based on their view of their mentee.

Results

This study failed to find a significant correlation between resident self-ratings of grit and those of their mentors.

Conclusions

The results of these two studies underscore the difficulty in obtaining accurate assessments of non-cognitive traits. These results further the understanding of the role of grit and raise important questions about how assessments might be used to assure validity. Further areas of inquiry into this potentially important characteristic are suggested.

## Introduction

Perhaps due to increasing concerns about stress, depression, and burnout in residents, there has been a recent focus on assessing and monitoring which are being called “non-cognitive” traits in residents and residency applicants [1, 2]. A similar situation exists in the world of sports where such attributes are often termed “intangibles,” obviously so named because they are difficult to measure and assess. Yet, much effort is expended in attempting to elucidate them because they are thought to be essential for performance and success. 

One such attribute that has received significant attention in medical training is that of grit, which is defined as trait-level perseverance and passion for long-term goals [3]. Grit has been found to predict tenacity in difficult situations and correlate with signs of burnout in medical populations. In a study of emergency medicine residents, grit scores correlated negatively (-.32) with emotional exhaustion and depersonalization (-.35) [4]. A study of surgical residents found that grit correlated positively (.30) with well-being and negatively (-.25) with both depression and (-.37) risk of attrition [5]. However, grit has not been well-studied in internal medicine residents.

There are challenges in utilizing non-cognitive attributes for the identification of ideal candidates, individuals at risk, monitoring of change in status and designing effective training or intervention. One obvious challenge is specifying what those important non-cognitive traits may be. A second critical challenge is how to measure those traits effectively and accurately. Measures with objective scoring systems (like the Grit scale) would seem advantageous [6].

However, even with objective evaluations, questions remain. One prime example is who should do the evaluation. Self-assessment has a long history and, assuming honesty and insight, should provide the most accurate personalized results. On the other hand, in residency programs, faculty are frequently called upon to make multiple evaluations of residents. When an objective measure is available, an important question is under what circumstances of administration is that measure reliable and accurate.

Olsen et al. conducted an important study that addressed this question related to grit in emergency medicine residents [7]. As part of a multi-center evaluation of a wellness curriculum, the authors sought to assess the congruence of resident self-evaluations of grit to ratings completed by residency faculty. In this study, 281 of 303 emergency residents completed the short grit scale (GRIT-S). Of these, 200 were able to have two faculty members of their choosing who they felt knew them well to complete the scale related to that resident.

The results indicated that while the inter-rater reliability between faculty members on a given resident was moderate, there was no statistically significant correlation between resident self-ratings and faculty ratings. Furthermore, faculty tended to overestimate grit levels compared to resident self-view. 

## Materials and methods

Objectives

The present study builds on the Olson study, but with residents from a different specialty, internal medicine. The goal was to ascertain whether internal medicine residents and their faculty mentors were congruent in their ratings of resident grit, or if not, how the ratings differed. Based on the previous work, it was hypothesized that faculty/mentor ratings and resident self-assessed ratings of grit would not be highly correlated and that faculty/mentors would over-estimate residents’ perceived levels of grit. 

Design

Subjects were internal medicine residents (N=42) in years one, two and three of their training at a community-based university-affiliated hospital internal medicine residency program. After obtaining IRB approval, residents completed the GRIT-S (short form) near the end of the 2018-2019 academic year. As each resident is assigned a mentor during their training, each resident’s mentor was also asked to complete the GRIT-S based on their view of their mentee. 

Twenty-six female and sixteen male residents completed the evaluation and were rated by one of eight mentors. There were 3 male residents and 15 female residents in year one; nine male residents and five female residents in year two; and four male residents and six female residents in year three. These characteristics are summarized in Table 1.

**Table 1:** Demographics of Participants

Residency Year	Male		Female		Total	
	Number	Percent	Number	Percent	Number	Percent
Year 1	3	16.67 %	15	83.33 %	18	42.86 %
Year 2	9	64.29 %	5	35.71 %	14	33.33 %
Year 3	4	40.00 %	6	60.00 %	10	23.81 %
Total	16	38.10 %	26	61.90 %	42	100.00 %

Measurements

Data from the GRIT-Scale (Short) was analyzed using the t-test and Pearson product-moment correlations.

## Results

**Figure 1:**
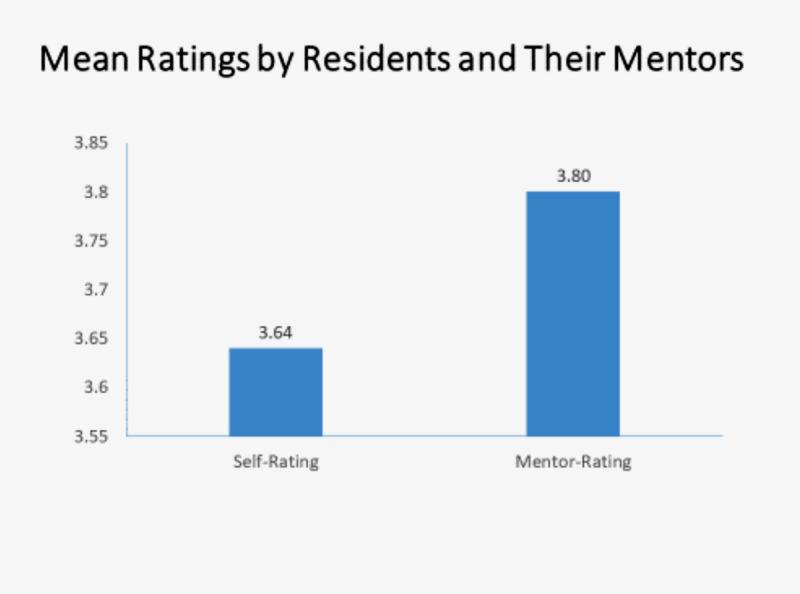
Mean Ratings by Residents and Their Mentors

The mean Grit-S score by resident self-rating was 3.64 (range 2.0 to 4.9) and the mean Grit-S score by mentor rating was 3.80 (range 1.8 to 4.8) as shown in Figure 1. The difference between these scores was not statistically significant (p< .28).

 

**Figure 2:**
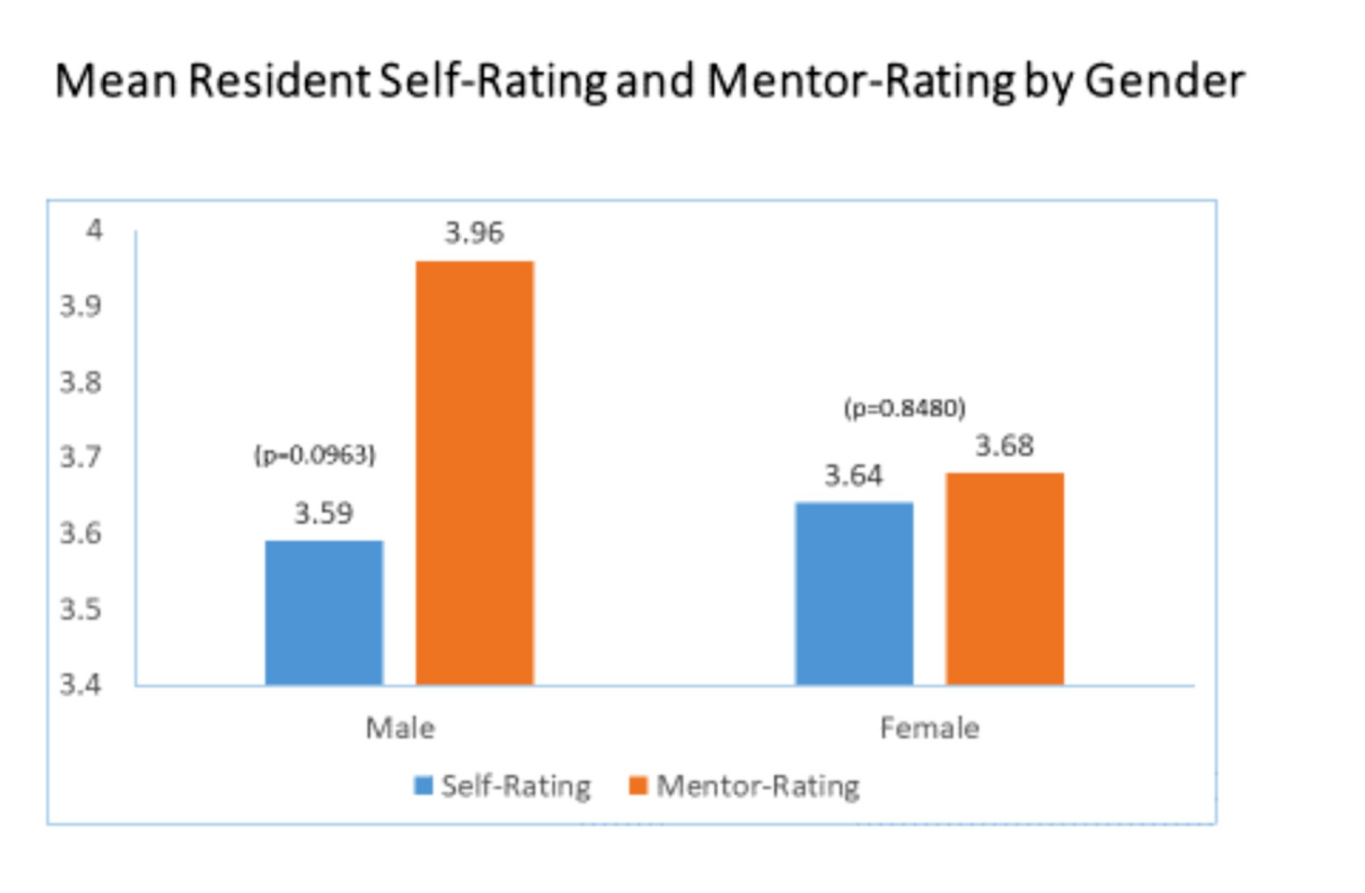
Mean Resident Self-Rating and Mentor Rating by Gender

While female residents self-rated grit higher than male residents, as did resident mentors, these differences were not statistically significant as shown in Figure 2. 

**Figure 3:**
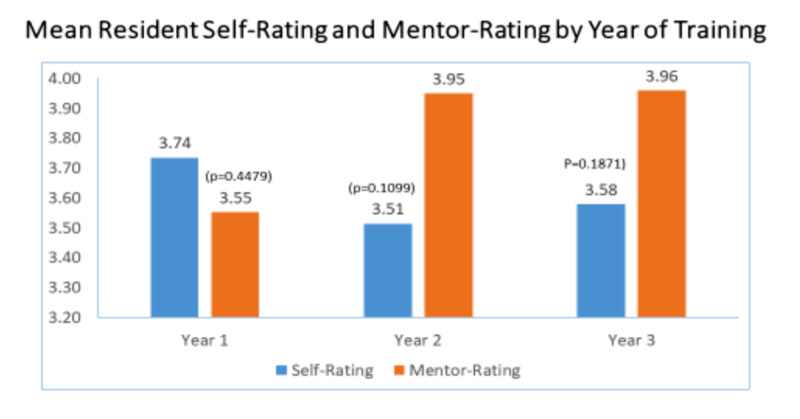
Mean Resident Self-Rating and Mentor-Rating by Year of Training

There was no significant difference in mean ratings by year of training (year 1 p<.45, year 2 p<.11, year 3 p<.19 (figure 3).

**Figure 4:**
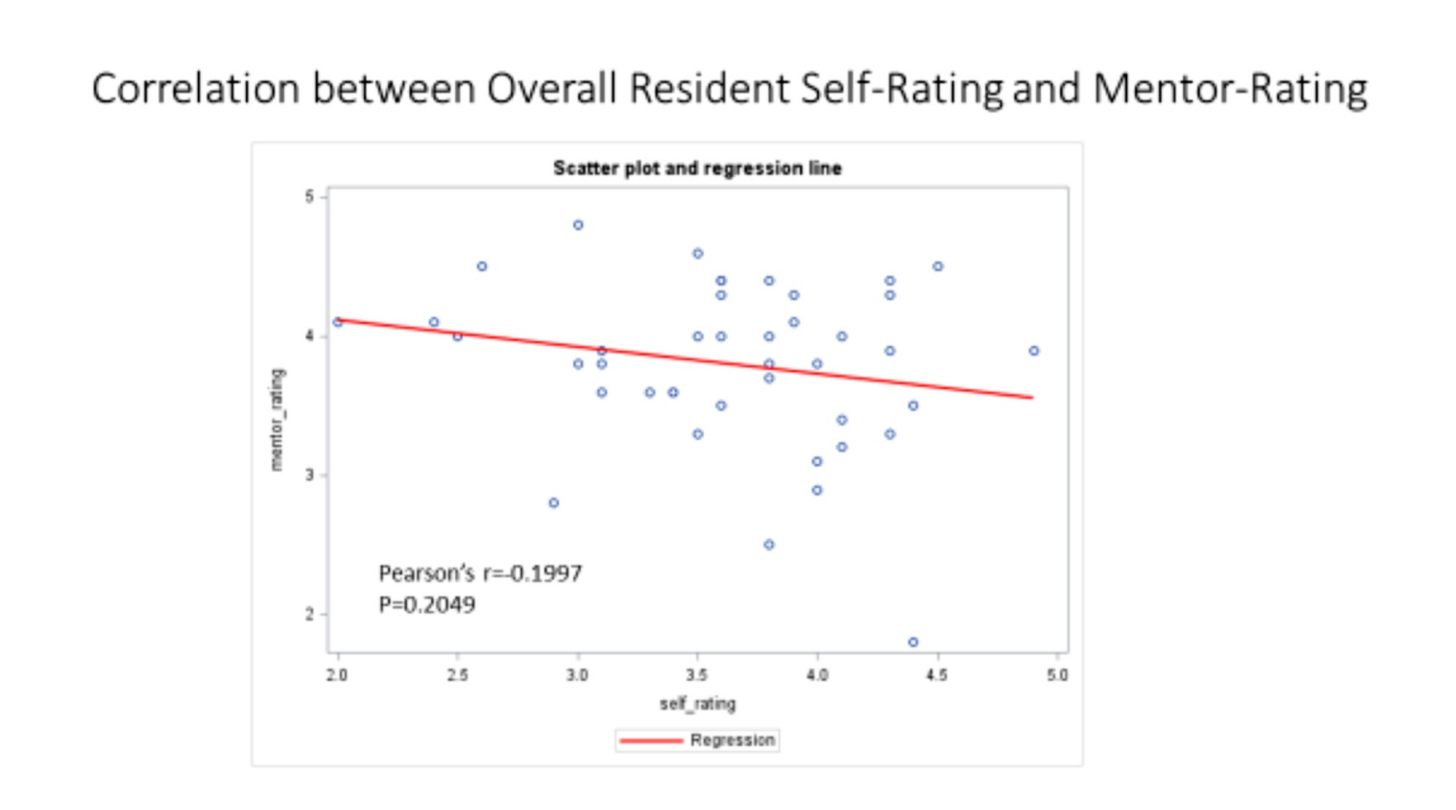
Correlation between Overall Resident Self-Rating and Mentor Rating

The correlation between self-ratings and mentor ratings was not significant r=0.1996 p<.20 as displayed in Figure 4.

Sixteen faculty ratings (38.1%) of resident grit were lower than what was self-rated by the resident and twenty-three faculty ratings (54.8%) were higher than resident self-ratings. The mean under-rating was 0.72 and the mean over-rating was 0.80. These means were not significantly different p<.7. Mentor and resident self-ratings were equivalent in three cases (7%).

## Discussion

Like the Olson study on emergency medicine residents, this study of internal medicine residents found no significant correlation between observer (mentor) ratings and resident self-ratings of the residents’ level of grit.

**Figure 5:**
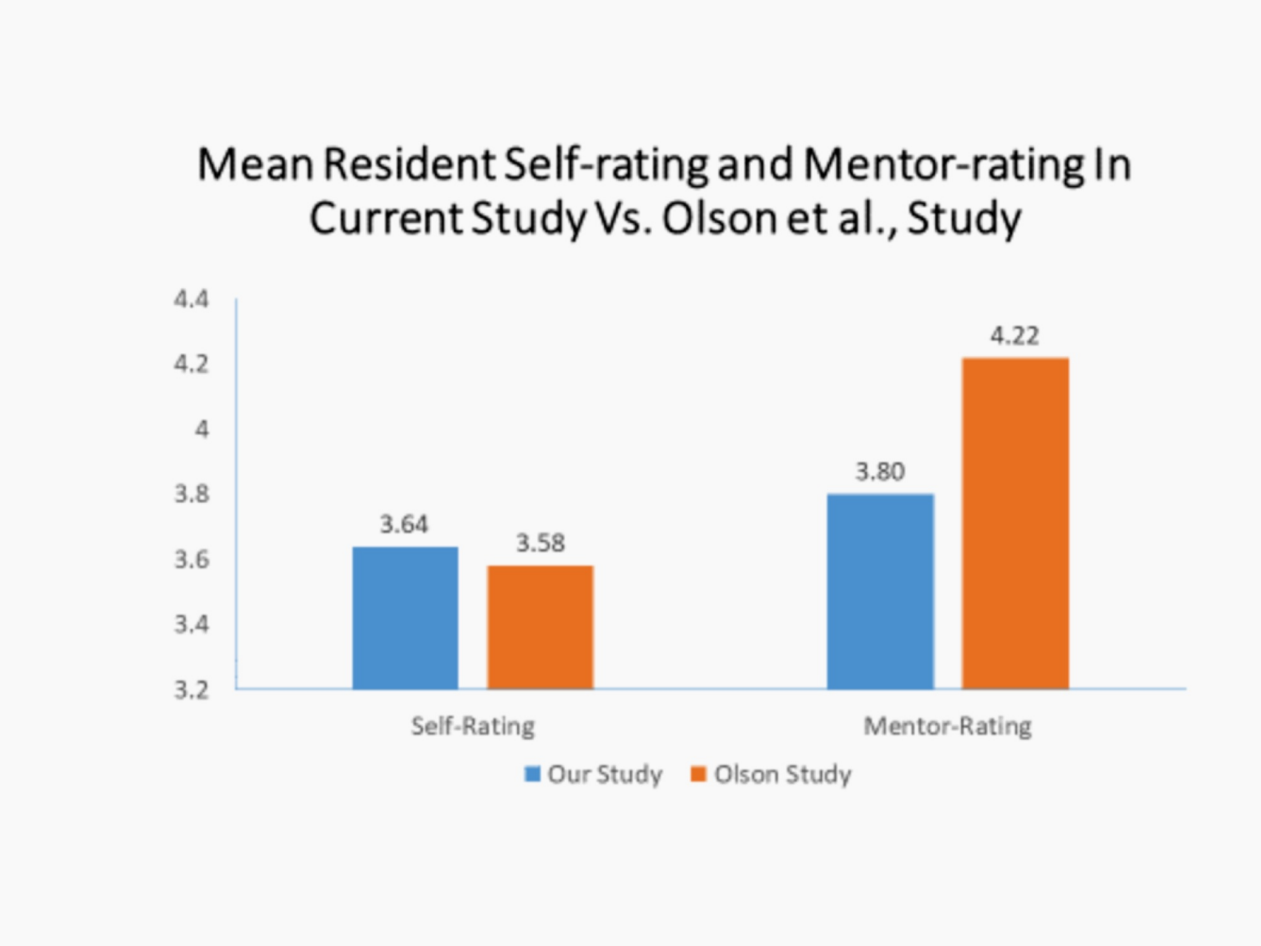
Mean Resident Self-Rating and Mentor-Rating in Current Study vs Olson et al. Study

Resident self-assessed levels of grit were similar in this study (3.64) compared to the Olson study (3.58) as shown in Figure 5. However, unlike that study where faculty grossly over-estimated grit levels of residents (4.22 vs 3.58), in the current study there was a much smaller difference between mentor-assessed resident grit levels and resident self-assessed grit levels (3.8 vs 3.64) as also shown in Figure 5.

The results of these two studies underscore the difficulty in obtaining accurate assessments of non-cognitive traits. The implication of the lack of correlation is not entirely clear. A similar lack of congruence between faculty and resident self-ratings of another important variable, burnout, has been documented [5] [8]. In various other areas of professional education, it appears that faculty also rate performance and personality characteristics more highly than students do themselves [9, 10]. Other work has concluded that physicians have a limited ability to accurately self-assess [11]. The concept of “social desirability”, a bias to respond in a socially expected manner or positive light (or the inverse of not wanting to appear overconfident or egotistical), is concerning in these types of evaluations [12].

Unfortunately, at this point, it is unknown which set of ratings (mentor/faculty or self-ratings) is more accurate and predictive. Further research linking scores to relevant outcomes is needed to clarify this issue. 

Grit has been demonstrated to measure a variable that seems to predict tenacity in difficult situations and has promising, if inconclusive value in medical education [12, 13]. Taken together, these studies further our understanding of the role of grit and raise important questions about how it might be used to assure validity and suggest further areas of inquiry outlined by the issues raised above. 

Limitations:

The present study differed from that of the Olson study in that it included a smaller number of residents representing only one institution. The small sample size reduces the power of the study making significant results less likely. Also, this study utilized mentors, rather than resident selected raters. 

The impact of using mentors, who should know their resident mentees fairly well, especially over three years, is also unclear and may have affected the results. The length of time being a mentor for a resident, i.e., how much knowledge a mentor has about a resident and the quality of their relationship may be an important factor. Unfortunately, the number of subjects in this study was not sufficient to address this. However, contrary to expectation, there appeared to be a trend of decreasing strength of association with the length of relationship. 

## Conclusions

While a congruence of ratings for characteristics like grit is desirable, a methodology of using both self and other ratings, especially with a discrepancy of scores does suggest a topic of potential discussion between resident and mentor/faculty. Discussing discrepancies can facilitate the relationship with a mentor, allow more accurate views of the resident, and, importantly, suggest need or type of intervention to strengthen a selected trait. 
